# Association Between Endocrine Diseases and Serous Otitis Media in Children

**DOI:** 10.4274/jcrpe.3585

**Published:** 2017-03-01

**Authors:** Murat Koçyiğit, Taliye Çakabay, Safiye G. Örtekin, Teoman Akçay, Güven Özkaya, Selin Üstün Bezgin, Melek Yıldız, Mustafa Kemal Adalı

**Affiliations:** 1 Kanuni Sultan Süleyman Training and Research Hospital, Clinic of Otolaryngology, İstanbul, Turkey; 2 Medical Park Gaziosmanpaşa Hospital, Clinic of Pediatric Endocrinology, İstanbul, Turkey; 3 Uludağ University Faculty of Medicine, Department of Biostatistics, Bursa, Turkey; 4 Kanuni Sultan Süleyman Training and Research Hospital, Clinic of Pediatric Endocrinology, İstanbul, Turkey; 5 Bir Nefes Private Hospital, Clinic of Otolaryngology, Kırklareli, Turkey

**Keywords:** Otitis media with effusion, endocrine diseases, tympanometry

## Abstract

**Objective:**

Otitis media with effusion (OME) is a condition in which fluid is retained in the middle ear cavity. The association between endocrine disorders and OME has not yet been determined. This study aimed to investigate the presence of OME in children diagnosed with an endocrine disease and the relationship between these two conditions.

**Methods:**

The study was conducted on 918 pediatric patients (440 boys, 478 girls; mean age: 8.40, range 3-15 years) and 158 healthy controls (76 boys, 79 girls; mean age: 8.31, range 3-15 years). All children underwent an ear examination and a tympanometry performed by an otorhinolaryngologist. Tympanometry results were used to diagnose OME.

**Results:**

OME was detected in 205 (22.3%) of 918 patients and in 19 (12.0%) of 158 subjects in the control group. The difference in frequency of OME between the two groups was statistically significant (p=0.003).

**Conclusion:**

The results of the study reveal that there may be a tendency towards the occurrence of OME in pediatric endocrinology patients.

**WHAT IS ALREADY KNOWN ON THIS TOPIC?**

Relationship between otitis media with effusion (OME) and endocrine diseases is not clear in pediatric population.

**WHAT THIS STUDY ADDS?**

Specific endocrine diseases such as metabolic syndrome, growth hormone deficiency, hypothyroidism, and idiopathic short stature may accompany OME.

## INTRODUCTION

Despite the development of antibiotics and advances in surgical techniques, the frequency of otitis media with effusion (OME) has been increasing (1). OME is a condition in which fluid is retained in the middle ear cavity but without otalgia, fever, or other symptoms (2). This condition has been shown to be caused by complex reactions involving the dysfunction of the Eustachian tubes, infection in the mucosa, immune deficiency, and allergy, among others (3). The incidence of OME varies widely, being reported as 50% in British children (4), 8.7% in Japanese children (5), 8% in Nigerian children (6) with differences according to surveyed regions. OME is a leading cause of hearing impairment in children, and its early and proper management can prevent hearing and speech impairment, which can cause developmental delay in children (7). The incidence of endocrine disorders has also been increasing, but the association between endocrine disorders and OME has not yet been determined. Although there are many studies in the literature about many diseases that are believed to be associated with OME, there are no comprehensive studies showing a relationship between endocrine diseases and OME. This study aimed to investigate the presence of OME in children diagnosed with an endocrine disease and the relationship of these conditions with one another.

## METHODS

Our study was conducted on 918 pediatric patients (440 boys, 478 girls; mean age: 8.40, range 3-15 years) who presented to the pediatric endocrinology outpatient clinic and on 158 children with no otolaryngologic or endocrine problems, constituting the healthy control group (76 boys, 79 girls; mean age: 8.31, range 3-15 years). All partipicants in the study group had at least one endocrine disease. Patients who had active otorhinolaryngological symptoms, ear wax, cleft palate repair history in the past, and cases with submucous cleft palate were excluded from the study. Children diagnosed with an endocrine or metabolic disease, who did not have any otorhinolaryngologic symptoms were compared to healthy children in the control group for presence of OME. The study was conducted from April to October 2015 due to the circulation of respiratory viruses during winter months. Informed consent was obtained from all individual participants included in the study or their parents.

A total of 918 patients in 24 different disease groups were evaluated. Those diseases with numbers of patients under 18 were excluded from the statistical evaluation; their OME rates were given only. The study was approved by the Institutional Review Board. Informed written consent was obtained from the parents of the children studied after explanation of the research purpose.

All children underwent a complete otolaryngologic examination. A flexible nasopharyngoscope was used to detect adenoid hypertrophy. Both ears were examined using an otoscope. Tympanometry was performed by an otolaryngologist using a MAICO m40, (Minneapolis, USA). Tympanometric measurement results were classified according to adjusted Jerger’s classification as types A, As, B, or C (8). Types A and As curves were accepted as no effusion in the middle ear, while types B and type C were considered as predictive of OME. Tympanometry results were used to diagnose OME.

Statistical analysis were done by using Statistical Package for the Social Sciences 22.0 operating program (license no: 10240642). Pearson chi-square test and Fisher-Freeman-Halton test were used. Significance limit was set at p<0.05.

## Results

When the 918 patients and 158 healthy children who participated in the study were compared in terms of their ages, a statistically significant difference was not detected (p=0.086). While 88 (9.6%) of the 918 patients had adenoid vegetation, 830 patients (90.4%) did not. However, when patients with adenoid vegetation were compared to those who did not have adenoid vegetation for presence of OME, a statistically significant difference was not observed (p=0.717). On the other hand, a comparison of OME incidence in the two groups revealed a statistically significant difference (Table 1). Due to their small numbers, patients with hypophyseal insufficiency, adrenal insufficiency, hyperthyroidism, hyperinsulinemia, diabetes insipidus, MEN-1 (multiple endocrine neoplasia), Graves’ disease, metabolic syndrome, macro-prolactinemia, hypoglycemia, vitamin D deficiency, hirsutism, congenital adrenal hyperplasia, hypogonadism, and primary amenorrhea were excluded from the statistical analysis (Table 2). OME ratios and the results of the comparisons in the remaining patients with the controls are shown in Table 3.

## DISCUSSION

OME is a leading cause of hearing impairment in children. Its early and proper management can avoid hearing and speech impairment and consequent developmental delay in children (7). Among the factors thought to influence the effects of OME are age, sex, race, season of the year, hereditary factors, number of family members, smoking status of parents, diseases experienced by children, and nursing methods. Factors reported to predispose to OME include upper respiratory tract infection, age, race, and attendance in day care centers, whereas factors that do not significantly influence OME include bronchitis, cystic fibrosis, socioeconomic status, smoking by parents, and antibiotic abuse (9). The association between endocrine disorders and OME has not yet been investigated. In our study, children diagnosed with an endocrine or metabolic disease and who did not have any otorhinolaryngologic symptoms were compared to healthy children in the control group for presence of OME.

In our study, a statistically significant difference was observed between the patient group and the controls in OME incidence, a finding suggesting that there may be a tendency towards the occurrence of OME in pediatric endocrine diseases.

In the relevant literature, while there is very little information about the importance of diabetes mellitus for ear diseases, the focus is on the impact of diabetes mellitus on patients with external otitis, malignant external otitis, otitis media, sudden sensorineural hearing loss and slowly progressive hearing loss (10).

Kim et al (11) assessed 140 children aged 2-7 years who underwent unilateral or bilateral ventilation tube insertion for treatment of OME (experimental group) and 190 children with no history of OME who underwent operations for conditions other than ear diseases during the same period and reported that childhood obesity was significantly higher in children with OME. This finding suggests that childhood obesity could have an effect on the development of OME. Kim et al (12) reported that pediatric obesity may have an effect on the development of OME, but pediatric overweight was not reported to be associated with occurrence of OME.

Middle ear problems are reported in rare genetic syndromes that cause short stature such as achondroplasia and cartilage-hair hypoplasia (13,14,15). However, we did not find a study investigating incidence of middle ear diseases in children with idiopathic short stature.

There is a study showing that hearing loss may occur in patients with congenital hypothyroidism, even if they receive sufficient treatment (16). Although thyroid hormone replacement therapy was adequate in the patients in our study, OME incidence was higher than in the control group.

Micro- and macro-nutrition deficiencies can occur in malnourished children. It was reported that vitamin D and zinc deficiencies impair the function of the Eustachian tube and lead to middle ear problems and that this situation can be improved with treatment (17,18,19). We did not find a significant difference in frequency of OME between patients with nutritional deficiencies and the control group. The reason for this may be that our patients suffered mild or moderate malnutrition rather than severe malnutrition.

In the study conducted by Bergamaschi et al (20), persistent secretory otitis media was detected in 21.3% of 173 patients with Turner syndrome. In our study, the incidence of OME in patients with Turner syndrome was similar to this result.

The results of our study indicate that there may be a tendency towards the occurrence of OME in pediatric endocrine diseases. We believe further studies on the relationships particularly of metabolic syndrome, hypothyroidism, growth hormone deficiency, and idiopathic short stature with OME might be beneficial.

## Figures and Tables

**Table 1:**
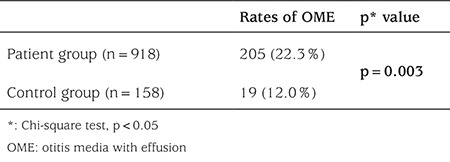
Comparison between the patient and control groups for otitis media with effusion

**Table 2:**
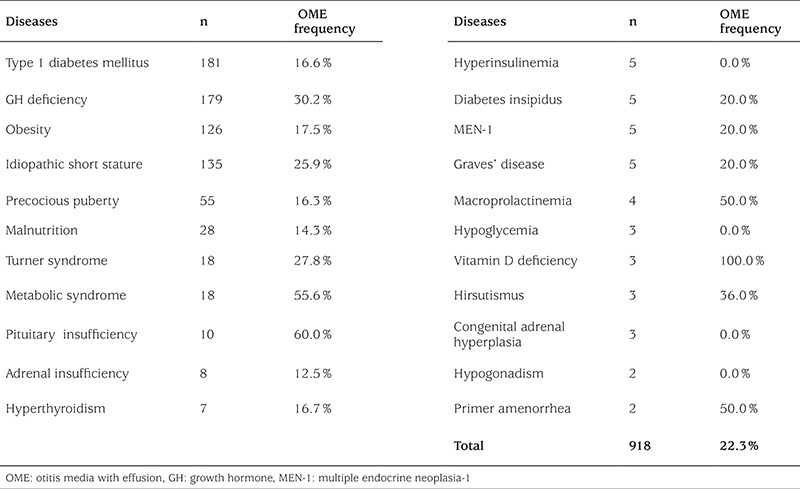
The numbers (n) of patients and otitis media with effusion frequency in endocrine patients included in the study

**Table 3:**
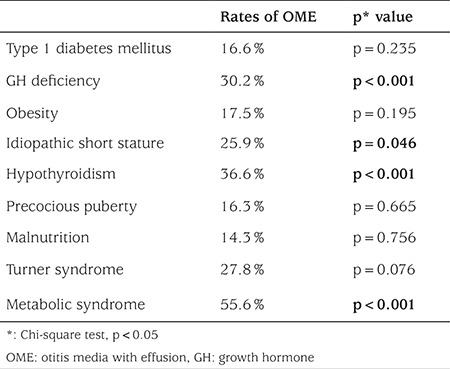
Rates of otitis media with effusion and comparisons between controls and otitis media effusion patients with endocrine diseases

## References

[1] Bluestone CD, Berry QC (1976). Concept on the pathogenesis of middle ear effusion. Ann Otol Rhinol Laryngol.

[2] Casselbrant ML, Brostoff  LM, Cantekin EI, Flaherty MR, Doyle WJ, Bluestone CD, Fria TJ (1985). Otitis media with effusion in preschool children. Laryngoscope.

[3] Ilicali OC, Keles N, Deger K, Savas I (1999). Relationship of passive cigarette smoking to otitis media. Arch Otolaryngol Head Neck Surg.

[4] Brooks DN (1976). School screening for middle ear effusion. Ann Otol Rhinol Laryngol.

[5] Morimoto K, Narita S, Kawaguchi E, Yamagishi M, Kataura A (1991). Epidemiological analysis of otitis media with effusion in children. Nihon Jibiinkoka Gakkai Kaiho.

[6] Ogisi FO (1988). Impedance screening for otitis media with effusion in Nigerian children. J Laryngol Otol.

[7] Yousaf  M, Inayatullah Khan F (2012). Medical versus surgical management of otitis media with effusion in children. J Ayub Med Coll Abbottabad.

[8] Jerger J (1970). Clinical experience with impedance audiometry. Arch Otolaryngol.

[9] Van Cauwenberge PB (1984). Relevant and irrelevant predisposing factors in secretary otitis media. Acta Otolaryngol Suppl.

[10] Avetisyan N, Lautermann J (2014). Ear diseases and diabetes mellitus. HNO.

[11] Kim JB, Park DC, Cha CI, Yeo SG (2007). Relationship between pediatric obesity and otitis media with effusion. Arch Otolaryngol Head Neck Surg.

[12] Kim SH, Park DC, Byun JY, Park MS, Cha CI, Yeo SG (2011). The relationship between overweight and otitis media with effusion in children. Int J Obes (Lond).

[13] Collins WO, Choi SS (2007). Otolaryngologic manifestations of achondroplasia. Arch Otolaryngol Head Neck Surg.

[14] Türkkani-Asal G, Alanay Y, Turul-Ozgür T, Zenker M, Thiel C, Rauch A, Unal S, Gürgey A, Tezcan I (2009). Mild clinical phenotype and subtle radiographic findings in an infant with cartilage-hair hypoplasia. Turk J Pediatr.

[15] Lyford-Pike S, Hoover-Fong J, Tunkel DE (2012). Otolaryngologic manifestations of skeletal dysplasias in children. Otolaryngol Clin North Am.

[16] Vanderschueren-Lodeweyckx M, Debruyne F, Dooms L, Eggermont E, Eeckels R (1983). Sensorineural hearing loss in sporadic congenital hypothyroidism. Arch Dis Child.

[17] Elemraid MA, Mackenzie IJ, Fraser WD, Brabin BJ (2009). Nutritional factors in the pathogenesis of ear disease in children: a systematic review. Ann Trop Paediatr.

[18] Cayir A, Turan MI, Ozkan O, Cayir Y, Kaya A, Davutoglu S, Ozkan B (2014). Serum vitamin D levels in children with recurrent otitis media. Eur Arch Otorhinolaryngol.

[19] Gulani A, Sachdev HS (2014). Zinc supplements for preventing otitis media. Cochrane Database Syst Rev.

[20] Bergamaschi R, Bergonzoni C, Mazzanti L, Scarano E, Mencarelli F, Messina F, Rosano M, Iughetti L, Cicognani A (2008). Hearing loss in Turner syndrome: results of a multicentric study. J Endocrinol Invest.

